# Associations of the perceived benefits and harms of COVID-19 with confidence in coping with the pandemic and mental health symptoms: a population-based survey in Hong Kong

**DOI:** 10.3389/fpubh.2023.1175085

**Published:** 2023-06-20

**Authors:** Ying Yao, Wei Jie Gong, Agnes Yuen Kwan Lai, Yongda Socrates Wu, Shirley Man Man Sit, Man Ping Wang, Sai Yin Ho, Tai Hing Lam

**Affiliations:** ^1^School of Nursing, LKS Faculty of Medicine, The University of Hong Kong, Pokfulam, Hong Kong SAR, China; ^2^Department of General Practice, Health Science Center, Shenzhen University, Shenzhen, Guangdong, China; ^3^School of Public Health, LKS Faculty of Medicine, The University of Hong Kong, Pokfulam, Hong Kong SAR, China

**Keywords:** COVID-19, perceived benefit, perceived harm, confidence, coping, mental health

## Abstract

**Introduction:**

Both perceived benefits and harms of COVID-19 have been reported, but whether they affect confidence in coping with the pandemic and mental health remains uncertain.

**Objective:**

To examine the association of perceived benefits and harms of COVID-19 with confidence in coping with the pandemic and mental health symptoms.

**Methods:**

A population-based survey was conducted on 7,535 Hong Kong adults from 22 February to 23 March 2021, when the 4^th^ wave of COVID-19 was under control. Information on sociodemographic characteristics, perceived benefits (10 options) and harms (12 options) of COVID-19, confidence in coping with the pandemic (range 0–10), loneliness (range 0–4), anxiety (General Anxiety Disorders-2, range 0–6) and depression (Patient Health Questionnaire-2, range 0–6) was collected. Latent profile analysis was used to identify the combined patterns of perceived benefits and harms of COVID-19. The associations of combined patterns with confidence in coping with COVID-19, loneliness, anxiety, and depression were examined using linear regression (β coefficient) adjusting for sociodemographic characteristics.

**Results:**

The combined patterns of perceived benefits and harms were classified into benefit (*n* = 4,338, 59.3%), harm (*n* = 995, 14.0%), and ambivalent (*n* = 2,202, 26.7%) groups. Compared with the ambivalent group, the benefit group had a significantly higher level of confidence (adjusted β 0.46, 95% CI 0.33 to 0.58), and lower levels of loneliness (−0.35, −0.40 to-0.29), anxiety (−0.67, 0.76 to-0.59), and depression (−0.65, −0.73 to-0.57). The harm group had a significantly lower level of confidence (−0.35, −0.53 to-0.16), and higher levels of loneliness (0.38, 0.30 to 0.45), anxiety (0.84, 0.73 to 0.96), and depression (0.95, 0.84 to 1.07).

**Conclusion:**

Perceived greater benefit from COVID-19 was associated with better mental health and stronger confidence in coping with the pandemic.

## 1. Introduction

The COVID-19 pandemic caused an unprecedented challenge to public health, including enormous loss of life worldwide and exacerbating psychological symptoms of anxiety, depression, and stress (1). Daily life had been severely affected by social restrictions, border controls, school closures, and stay-at-home orders (2). The unemployment rate reported by the Organization for Economic Cooperation and Development (OECD) rose by 3% and reached 8.8% at the onset of the outbreak, the highest rate in a decade (3). In contrast, potential benefits of the pandemic included increased hygiene literacy and stringent control measures, followed by plummeted global cold and flu cases (4, 5). Long-term work-from-home arrangements amid the pandemic also improved family relationships, communication, and emotional expression (6).

Individuals’ positive and negative perceptions of the pandemic can coexist with independence (7). Three groups of perceived benefits and harms of COVID-19 were identified at the beginning of the outbreak (data as of May 2020): indifferent group (both low perceived benefits and harms), harm group (high perceived harms but low benefits) and benefit group (high perceived benefits but low harms) (8). However, perception toward COVID-19 could change over time, given that people could be protected against the severe COVID-19 outcomes owing to the vaccine availability (9, 10), as well as their emotional adaption to the changes in the way of life and work brought by the pandemic (11). Our later survey (conducted from February to March 2021) found that perceived benefits increased substantially over time, while perceived harms were lower and stable (12), suggesting the grouping patterns of perceived benefits and harms may be changed.

Mental health crisis were emerging worldwide with COVID-19 outbreaks (13). People with higher perceived harms (such as reduced social interaction due to social distancing and quarantine, and perceived risk of infections) were more likely to have mental health symptoms (14, 15). In contrast, people who perceived benefits of COVID-19 reported better well-being (16). The identification and acknowledgment of benefits from negative experiences enable better coping with these negative situations (17). Assessing the mental health and confidence in coping with the pandemic in different groups could help identify at-risk populations and develop tailored interventions to address and alleviate widespread mental health concerns and enhance public confidence to cope with the pandemic, but no related literature was found.

In the present study, we first identified the grouping patterns of perceived benefits and harms of COVID-19 using data collected from February to March 2021, and then assessed the confidence in coping with the pandemic and mental health symptoms (loneliness, anxiety, and depression) in different groups.

## 2. Theory and literature review

The meaning-making theory posits that when faced with a stressful event, individuals reappraise and make meaning to the situation, possibly leading to negative and positive reframing (18), also known as perceived benefits and harms. Perceived harms from the COVID-19 pandemic and corresponding containment measures were common and widespread, mainly including increased psychological disorders, reduced income, and increased family conflicts (16, 19). In recent years, plenty of studies in the field of positive psychology had focused on the personal and environmental resources that can be mobilized or developed to cope with stressful events (18, 20, 21). Among these resources, benefits finding is defined as the identification or perception of benefits from adversities (22). Both perception and active seeking of benefits from stressful events were identified as cognitive reappraisal strategies that promote personal well-being (23, 24). Benefits from the COVID-19 pandemic mainly includes personal and relational levels, such as improved personal hygiene, increased rest time, and better family relationship (8).

Although perceived harm and benefit appear to represent mutually exclusive extremes of reframing valence, the existence of a positive reaction to a negative event does not mean that the negative impact is thereby eliminated (21). The dual attitudes theory model also assumes that negative and positive evaluations of the same object can co-exist independently (7), in a manner that mutually overrides rather than substitutes for each other, which could generate four possible grouping patterns: high perceived benefits and harms (ambivalent group); high perceived benefits and low harms (benefit group); high perceived harms and low benefits (harm group), and low perceived benefits and harms (indifferent group). Using latent profile analysis, three groups (indifferent, benefit, and harm) of perceived benefits and harms of COVID-19 (8) had been identified at the beginning of the outbreak. Grouping patterns of perceived benefits and harms changed over time (12), so re-exploration of grouping patterns is warranted.

COVID-19 and containment measures caused unprecedented and remarkable economic and health concerns. People with higher perceived harms from negative situations are prone to have psychological disorders, while those with higher perceived benefits had been linked to optimal adaptation to stressful events (25). However, no studies have explored the associations of different perceptions of COVID-19, a long-term and widespread stressful event, with mental health symptoms and confidence in coping with the pandemic. In this paper, we aim to address the following questions:

Q1: Have the pattern of perceived benefits and harms of COVID-19 changed as the outbreak progressed?

Q2: What are the mental health status and confidence in coping with pandemic among people with different perceptions of COVID-19?

## 3. Materials and methods

### 3.1. Study design

Under the Hong Kong Jockey Club Smart Family-Link Project, we conducted the Family Amidst COVID-19 2 (FamCov-2) survey using a population-based combined sampling frame of landline telephone, mobile telephone, and online surveys on Hong Kong residents aged 18 and above (*n* = 7,535) from February 22 to March 23, 2021, when the fourth wave of the COVID-19 pandemic was under control in Hong Kong. We had three subsets of questions for FamCov-2 with each consisting of the core questions and the subset-specific questions to avoid burdening the respondents. About one-third of the respondents randomly answered the subset-specific questions. Finally, 7,535 respondents provided data on perceived benefits and harms of COVID-19, mental health, and social demographic characteristics. Four thousand six hundred sixty-two respondents provided data on confidence in coping with the pandemic.

The study was carried out in accordance with the guidelines and regulations set out in the Declaration of Helsinki. Ethical approval was granted by the Institutional Review Board (IRB) of the University of Hong Kong Hospital Authority Hong Kong West Cluster (UW 20–651). Informed consent was obtained from all respondents before the survey.

### 3.2. Sampling methods

Detailed methods and procedures have been reported elsewhere (12, 26). Briefly, the landline and mobile telephone interviews were conducted by well-trained interviewers from Hong Kong Public Opinion Research Institute (HKPORI), a well-known local survey agency. All phone numbers were randomly drawn from a phone list that was generated by using known prefixes assigned to telecommunication service providers. Invalid numbers were deleted. Each telephone interview took around 10 min. For the landline telephone survey, only one eligible respondent (whose next birthday is nearest to the interview date) was selected in a household. Among 1,604 and 816 valid landline and mobile telephone samples, 1,022 (63.7%) and 500 (61.2%) respondents completed the interview, respectively. For the online survey, email invitations were sent by HKPORI to their probability and non-probability online panels. Of 4,311 and 44,514 probability and non-probability group members who opened the email, 641 (14.2%) and 5,372 (12.1%) respondents completed the survey, respectively.

### 3.3. Measurements

Perceived benefits of COVID-19 were asked by a question “what benefits have the COVID-19 outbreak brought to you?” with 10 options: improved general health; improved individual hygiene; decreased colds; decreased negative emotions; increased positive emotions; increased ability to cope with difficulties; improved efficiency of work/study at home; increased private time; increased rest time; increased knowledge of epidemic prevention. Perceived harms of COVID-19 were asked by a question “what harms have the COVID-19 outbreak brought to you?” with 12 options: increased physical pain; gained weight; decreased physical activities; worse sleep quality than before; increased mental distress; increased negative emotions; caused depression; caused anxiety; decreased efficiency of work/study from home; decreased private time; decreased rest time; delayed to see a doctor. Multiple options could be selected by respondents. There was no validated questionnaire on the perceived benefits and harms of COVID-19. The corresponding questions were designed by our team and have been published elsewhere (8, 12, 16). We had done some pilot tests, and no difficulties or sensitive issues were reported by pilot respondents, suggesting face validity.

Confidence in coping with the pandemic was assessed by a question “how much confidence do you have to deal with the COVID-19 pandemic.” The question was rated from 0 (not confident at all) to 10 (very confident). Loneliness was assessed by a question “in the past 7 days, how long have you been lonely?” with 5 options: None of the time (0 points); 1 to 2 days (1 point); 3 to 4 days (2 points); 5 to 6 days (3 points); 7 days (4 points). The four-item Patient Health Questionnaire (PHQ-4) was used to assess anxiety and depression symptoms in the past 2 weeks, which consists of the two-item General Anxiety Disorder (GAD-2) and the two-item Patient Health Questionnaire (PHQ-2) (27, 28). GAD-2 measured social panic and anxiety disorders, two core criteria for generalized anxiety with a Likert-like scale ranging from 0 (not at all) to 3 (nearly every day). PHQ-2 covered two core diagnostic criteria for depressive disorder, depressed mood, and loss of interest, with the same scoring method. Both GAD-2 and PHQ-2 scores range from 0 to 6, with a score of 3 or above suggesting anxiety and depression symptoms (29, 30). We had validated the Chinese version of the PHQ-2 in Hong Kong (31). The internal consistency of GAD-2 and PHQ-2 were 0.81 and 0.76, respectively, in the present study.

Information on sociodemographic characteristics collected included: sex, age (18–24; 25–44; 45–64; ≥65 years), education attainment (secondary/below; tertiary/above), household monthly income (HK$ < 10,000; 10,000-39,999; ≥40,000; HK$7.8 = US$1), number of cohabitants (0; 1–3; ≥4) and housing type (rented; owned). Socioeconomic status (SES) was calculated as a composite score using the sum of education (secondary or below, postsecondary), household monthly income per person (lower, higher, compared with the Hong Kong median income), and housing type (rented, owned), and was categorized as low, middle and high.

### 3.4. Statistical analysis

All statistical analyzes were performed using Stata 15.1 (StataCorp LLC, College Station, TX, United States). All data were weighted by sex, age group, and educational attainment of the Hong Kong general population in 2019 (32) to improve the representativeness of the sample. Latent profile analysis (LPA) was used to identify the combined patterns of perceived benefits and harms. LPA is a person-centered classification method that divides individuals into different subgroups based on similar characteristics. To determine the optimal number of profiles, the following model fit indices were considered: (a) lower Akaike information criterion (AIC); (b) lower Bayesian information criterion (BIC); (c) a minimal observed subgroup proportion of 5.00% or more; (d) classification accuracy, in which higher entropy is preferred; and (e) a comparison between k and k-1 profile models using Lo–Mendel–Rubin likelihood ratio tests (LMR) and bootstrap likelihood ratio test (BLRT), with *p* value less than 0.05 indicating preferred K-1 model. After determining the number of profiles, respondents were assigned to the most likely profiles based on the highest posterior membership probability. Chi-squared test was used to examine the sociodemographic differences in the different groups identified. With the ambivalent subgroup as a reference, multivariate logistic regression yield adjusted risk ratio (RR) and 95%CI confidence interval (CI) of the groups of perceived benefits or harms for sociodemographic characteristics with mutual adjustment. Multiple linear regression was used to calculate the adjusted β coefficient to examine the associations of the identified groups with confidence in coping with the pandemic, loneliness, anxiety, and depression.

## 4. Results

Table 1 shows that after weighting, 52.2% were female, 38.6% were aged 45 to 64 years, 64.9% had secondary or below educational attainment, and 49.6% had a household monthly income of HK$10,000 to 39,999 (US$1 = HK$7.8). 75.8% lived with 1 to 3 cohabitants and 58.5% lived in their owned housing. 58.6% had lower income compared to the median income in Hong Kong, and 57.3% had lower socioeconomic status. 26.7% (2,202/7535), 59.3% (4,338/7535) and 14.0% (995/7535) of respondents were categorized as ambivalent, benefit and harm groups, respectively, quite similar to unweighted results (29.2%, 57.6%, and 13.2%, respectively).

**Table 1:** Characteristic of the survey sample (*N* = 7,535).

	*n*	Unweighted (%)	Weighted^a^ (%)
Sex			
Male	3,635	48.5	47.8
Female	3,861	51.5	52.2
Age (years)			
18–24	589	7.9	8.8
25–44	3,026	40.5	32.7
45–64	2,884	38.5	38.6
≥65	982	13.1	20.0
Educational attainment			
Secondary/below	2,103	28.3	64.9
Tertiary/above	5,340	71.8	35.19
Household monthly income (HK$, US$1 = HK$7.8)			
<10,000	751	11.7	14.79
10,000-39,999	2,458	38.2	49.6
≥40,000	3,231	50.2	35.7
Number of cohabitants			
0	684	9.1	8.4
1–3	5,772	76.6	75.8
≥4	1,079	14.3	15.9
Housing type			
Rented	2,886	38.8	41.5
Owned	4,562	61.3	58.5
Household monthly income^b^			
Low	2,857	44.4	58.6
High	3,583	55.6	41.4
Socioeconomic status (SES)^c^			
Low (1)	2,134	33.3	57.3
Medium (2)	2,181	34.0	27.9
High (3)	2097	32.7	14.8
Patterns of perceived benefits and harms^d^			
Ambivalent group	2,202	29.2	26.7
Benefit group	4,338	57.6	59.3
Harm group	995	13.2	14.0

Sample size varied due to missing data.

^a^Results were weighted by sex, age, and education of the Hong Kong general population in 2019.

^b^Household monthly income was compared to HK median income.

^c^Socioeconomic status (SES) was calculated as a composite score using the sum of educational attainment (0 = secondary or below, 1 = postsecondary), household monthly income per person (0 = lower, 1 = higher, compared with the Hong Kong median income), and housing type (0 = rented, 1 = owned), and was categorized as low (0–1), middle (2) and high (3).

^d^The patterns of perceived benefits and harms of COVID-19 used the three profiles model identified by latent profile analysis.

**Table 2:** Results of statistical fit indices of latent profile analysis models (*N* = 7,535).

Model	AIC^a^	BIC^a^	aBIC^a^	LMR_P^b^	BLRT_P^b^	Entropy^c^	Composition^d^
One profile	67387.56	67415.27					
Two profiles	66931.95	66980.44	66958.20	<0.001	<0.001	0.71	17%/83%
Three profiles	65655.34	65724.61	65692.84	<0.001	<0.001	0.77	50%/32%/18%
Four profiles	66593.91	66683.96	66642.65	1.00	1.00	0.75	11%/32%/44%/12%

^a^Smaller Akaike information criterion (AIC), Bayesian information criterion (BIC), and sample-adjusted Bayesian information criterion (aBIC) suggest better model fitness.

^b^Lo–Mendel–Rubin likelihood ratio tests (LMR) and bootstrap likelihood ratio test (BLRT) were used to compare the K and K-1 profile models, with *p* < 0.05 indicating the preferred K-1 model.

^c^Entropy was computed to determine the accuracy of profile classification, with higher values indicating a better separation between profiles.

^d^The composition represents the percentage of each subgroup, with a minimum observed group size of 5% or more indicating relatively equal group allocation.

### 4.1. Latent profile analysis model identification

The results for the statistical fit indices of the LPA models recommend three profile patterns, given the low AIC and BIC, high entropy, and a relatively equal proportion assignment (50%, 32%, and 18%; Table 2). According to the scores of perceived benefits and harms of COVID-19, we labeled the three profiles as (a) the ambivalent group, with respondents reporting high scores in both benefits (mean = 3.51) and harms (mean = 4.19); (b) the benefit group with high scores in benefits (mean = 3.38) and low scores in harms (mean = 1.33); (c) the harm group with low scores in benefits (mean = 1.09) and high scores in harms (mean = 6.56).

Table 3 shows that more females were in the ambivalent group (29.5% vs. 23.7%) and harm group (15.1% vs. 12.8%) than males (*p* < 0.001). More respondents aged 65 years or above were in the benefit group (71.3%), and more aged 25 to 44 years were in the harm group (18.1%, *p* < 0.001). More respondents living in rental housing (14.4%, *p* = 0.02) or of low SES (15.6%, *p* = 0.02) were in the harm group.

**Table 3:** The patterns of perceived benefits and harms by weighted socioeconomic characteristics (*N* = 7,535).

	Ambivalent n (weighted %)	Benefit *n* (weighted %)	Harm *n* (weighted %)	*P*
Sex				
Male	970 (23.7)	2,190 (63.5)	475 (12.8)	
Female	1,227 (29.5)	2,118 (55.4)	516 (15.1)	<0.001
Age (years)				
18–24	208 (36.4)	287 (47.0)	94 (16.6)	
25–44	1,003 (29.2)	1,531 (52.7)	492 (18.1)	
45–64	784 (26.2)	1759 (61.4)	341 (12.4)	
≥65	198 (19.5)	723 (71.3)	61 (9.2)	<0.001
Educational attainment				
Secondary/ below	531 (25.9)	1,334 (59.3)	238 (14.8)	
Tertiary/above	1,647 (28.1)	2,951 (59.5)	742 (12.4)	0.07
Household monthly income (HK$, US$1 = HK$7.8)				
<10,000	175 (19.7)	479 (64.5)	97 (15.8)	
10,000-39,999	723 (26.5)	1,394 (58.0)	341 (15.5)	
≥40,000	975 (28.1)	1847 (61.2)	409 (10.7)	<0.001
Household monthly income compared to HK median income				
Low	801 (28.0)	1,661 (58.1)	395 (13.8)	
High	1,072 (29.9)	2059 (57.5)	452 (12.6)	0.15
Number of cohabitants				
0	186 (27.2)	412 (60.2)	86 (12.6)	
1–3	1,692 (29.3)	3,317 (57.5)	763 (13.2)	
≥4	324 (30.1)	609 (56.4)	146 (13.5)	0.08
Housing type				
Rented	866 (30.0)	1,605 (55.6)	415 (14.4)	
Owned	1,320 (28.9)	2,669 (58.5)	573 (12.6)	0.02
SES				
Low (0–1)	577 (24.9)	1,262 (59.5)	295 (15.6)	
Medium (2)	651 (27.7)	1,265 (60.7)	265 (11.6)	
High (3)	637 (27.1)	1,179 (61.7)	281 (11.2)	0.02

Sample size varied due to missing data.

Socioeconomic status (SES) was calculated as a composite score using the sum of educational attainment (0 = secondary or below, 1 = postsecondary), household monthly income per person (0 = lower, 1 = higher, compared with the Hong Kong median income), and housing type (0 = rented, 1 = owned), and was categorized as low (0–1), middle (2) and high (3).

### 4.2. Associations of the different perceptions of COVID-19 with confidence in coping with pandemic and mental health symptoms

Table 4 shows that, in the adjusted model, male (RR 1.30, 95% CI 1.16 to 1.46, *p* < 0.001) and the older (65 years or older: 2.54, 1.91 to 3.39, *p* < 0.001) had higher RR of the benefits. Those aged 65 or older (0.61, 0.39 to 0.95, *p* = 0.03) and with higher household monthly income (≥ HK$ 40,000: 0.55, 0.38 to 0.79, *p* = 0.001; HK$ 10,000-39,999: 0.67, 0.49 to 0.90, *p* = 0.01) had lower RR of the harms.

**Table 4:** Adjusted risk ratios (RR) of perceived benefits and harms by demographic characteristics (*N* = 7,535).

	Benefit vs. Ambivalent (Ref)		Harm vs. Ambivalent (Ref)	
	RR^a^	*P*	RR^a^	*P*
Sex				
Female	1		1	
Male	1.30 (1.16, 1.46)	<0.001	1.20 (1.02,1.42)	0.03
Age (years)				
18–24	1		1	
25–44	1.12 (0.90,1.40)	0.28	1.17 (0.86,1.58)	0.31
45–64	1.61 (1.28,2.02)	<0.001	0.97 (0.70,1.34)	0.86
≥65	2.54 (1.91,3.39)	<0.001	0.61 (0.39,0.95)	0.03
Educational attainment				
Secondary/below	1		1	
Tertiary/above	0.85 (0.70,1.04)	0.12	0.89 (0.67,1.18)	0.41
Household monthly income (HK$, US$1 = HK$7.8)				
<10,000	1		1	
10,000-39,999	0.92 (0.74,1.14)	0.46	0.67 (0.49,0.90)	0.01
≥40,000	0.92 (0.71,1.19)	0.54	0.55 (0.38,0.79)	0.001
Number of cohabitants				
0	1		1	
1–3	1.09 (0.87,1.37)	0.45	1.40 (0.98,2.00)	0.07
≥4	1.12 (0.85,1.49)	0.42	1.45 (0.95,2.23)	0.09
Housing type				
Rented	1		1	
Owned	0.98 (0.82,1.18)	0.87	0.82 (0.63,1.08)	0.15
SES				
Low (0–1)	1		1	
Medium (2)	1.09 (0.87,1.36)	0.46	0.96 (0.70, 1.33)	0.82
High (3)	1.07 (0.76, 1.50)	0.71	1.26 (0.78,2.06)	0.35

^a^Sex, age, educational attainment, monthly household income, number of cohabitants, housing type and SES were mutually adjusted.

Table 5 shows that the benefit group had a higher level of confidence in coping with the pandemic (β-coefficient 0.46, 95% CI 0.33 to 0.58, *p* < 0.001), and lower levels of loneliness (−0.35–0.40 to-0.29, *p* < 0.001), anxiety (−0.67, −0.76 to-0.59, *p* < 0.001), and depression symptoms (−0.65, −0.73 to-0.57, *p* < 0.001) adjusting for sociodemographic characteristics. The harm group had a lower level of confidence (−0.35, −0.53 to-0.16, *p* < 0.001), and higher levels of loneliness (0.38, 0.30 to 0.45, *p* < 0.001), anxiety (0.84, 0.73 to 0.96, *p* < 0.001), and depression symptoms (0.95, 0.84 to 1.07, *p* < 0.001).

**Table 5:** Association of perceived benefits and harms of COVID-19 with confidence in coping with the pandemic, loneliness, anxiety and depression symptoms (*N* = 4,662).

	Mean ± SD	Crude *β* (95% CI)	*P*	Adjusted^a^ *β* (95% CI)	*P*
Confidence in coping with pandemic (score 0–10)					
Ambivalent	6.59 ± 1.81	1		1	
Benefit	7.00 ± 1.77	0.44 (0.32, 0.55)	<0.001	0.46 (0.33, 0.58)	<0.001
Harm	6.27 ± 2.12	−0.37(−0.54, −0.20)	<0.001	−0.35(−0.53, −0.16)	<0.001
Loneliness (score 0–4)					
Ambivalent	0.91 ± 1.06	1		1	
Benefit	0.54 ± 0.82	−0.37(−0.42, −0.32)	<0.001	−0.35(−0.40, −0.29)	<0.001
Harm	1.36 ± 1.18	0.41 (0.33, 0.48)	<0.001	0.38 (0.30, 0.45)	<0.001
Anxiety (score 0–6)					
Ambivalent	4.01 ± 1.60	1		1	
Benefit	3.20 ± 1.33	−0.78(−0.86, −0.71)	<0.001	−0.67(−0.76, −0.59)	<0.001
Harm	4.88 ± 1.78	0.88 (0.76, 0.99)	<0.001	0.84 (0.73,0.96)	<0.001
Depression (score 0–6)					
Ambivalent	3.80 ± 1.52	1		1	
Benefit	3.04 ± 1.31	−0.76(−0.83, −0.68)	<0.001	−0.65(−0.73, −0.57)	<0.001
Harm	4.85 ± 1.77	0.97 (0.86, 1.08)	<0.001	0.95 (0.84, 1.07)	<0.001

^a^Adjusted for sex, age, educational attainment, monthly household income.

## 5. Discussion and conclusion

### 5.1. Principal findings

We found that respondents who perceived greater benefits from COVID-19 had better mental health and greater confidence in coping with the pandemic, those who perceived greater harms had poorer mental health and lower confidence and were thus at greater risk. Our finding shows consistency with the insight that personal reappraisal of an event can generate both negative and positive reframing, as posited by meaning-making theory. Perceived negative impact of COVID-19 on lifestyle behaviors has been reported as a risk factor for mental health symptoms (33–35). Mental health symptoms, in turn, also led to various unhealthy behaviors (unhealthy eating habits, sleep disturbances, and increased substance use) (36–38), forming a vicious cycle. The potential risk of individuals perceiving great harm should be highlighted, and interventions are needed to facilitate their positive adjustment.

The three groups (ambivalent, benefit, and harm) identified by LPA, confirmed the independence of benefits and harms suggested by the dual attitude theoretical model. This pattern differed from those in our previous FamCov-1 survey (indifferent, benefit, and harm) (8), possibly reflecting the substantial increase in perceived benefits from COVID-19 and no large decrease in perceived harms from the FamCov-1 to the FamCov-2 survey (12). Through repeated outbreaks of COVID-19, people gradually adapted and built resilience to cope with stressful events, which explained the increased number of people who perceived benefits. Conversely, the threat of infection and continuing stringent control measures (quarantine, isolation, and restriction of cross-border travel) resulted in consistently high levels of perceived harms.

More females were found in the ambivalent group and harm group. Females were more prone to fear and perceived a greater threat from stressful events than males (39, 40). Females who were employed and worked from home amid the pandemic reported greater work–family conflict (41) and augmented burnout symptoms (42) than males. Some females such as housewives were less directly affected by job loss, working from home, and unpaid leave due to the pandemic. Unexpectedly, more respondents aged 45–65 and over 65, who might be more vulnerable and had more chronic diseases, were more likely to perceive the benefits of COVID-19, probably because they had more experience (such as the past experience during the SARS epidemic in Hong Kong in 2003) and knowledge to deal with difficulties and were better able to adapt to adversity. Respondents with higher monthly household incomes should have stronger financial support to cope with economic challenges from the pandemic and therefore perceived less harms from COVID-19.

Respondents in the harm group were less confident in coping with the pandemic. Since more respondents with low SES were in the harm group, they might have or perceive a greater economic threat amid the pandemic, and the increased uncertainty and fearful feeling may lead to lower confidence in coping with the pandemic. Confidence in coping might influence people’s coping strategies. A previous survey in Taiwan found that having sufficient basic protective equipment, financial support, medical resources, and higher levels of social support were associated with higher levels of confidence in coping with the pandemic (43). Additional support and assistance for people in the harm group are needed to build their confidence in coping with difficulties amid the COVID-19 pandemic.

Consistent with previous studies reporting that the perceived impact of COVID-19 on daily life was associated with greater psychological consequences (44, 45), we found that respondents in the harm group showed more loneliness, anxiety and depressive symptoms. Concerns about current terrible situations and future negative consequences of COVID-19 could cause various mental and psychological symptoms (46, 47). A positive perspective can lead to positive emotions, adaption, and positive growth even in the face of threats and challenges (48). Cognitive reappraisal, an emotion regulation strategy in which people perceive stressful events as positive challenges rather than just negative threats (49), has been linked to decreased perceived anxiety and stress symptoms (50).

### 5.2. Implications

Positive psychological emotions are important to the psychological recovery process in individuals who experience intense stressors and mental disorders. Identifying and acknowledging the potential benefits of stressful events is linked to coping in a positive manner (22). It is suggested that while reporting the negative effects of the pandemic, governments and health agencies should advocate for the public to change their perspective and find potential benefits behind the negative effects of the pandemic.

Our findings highlight the need to identify and assist people in the harm group. Promoting positive emotions and adaptive coping skills can help minimize negative psychology. Educational or training program on cognitive reappraisal should be developed and delivered to the public (especially the harm group) to alter their negative appraisals, and build positive psychological resources to avoid psychological consequences.

### 5.3. Limitations

Our study had some limitations. First, causal inferences could not be drawn in this cross-sectional study. Second, recall bias might exist as all data were self-reported. However, collecting self-reported data from such a large sample using actual non-face-to-face way was practicable way amid the pandemic. Third, to minimize the length of the questionnaire and reduce the burden on the respondents, we did not collect more data on the intensity of perceived benefits and harms. Fourth, classification error might occur as respondents were assigned to specific profiles based on the posterior probabilities without knowledge of actual affiliation, but LPA is a robust and human-centered classification method. Finally, our results may not be generalizable to other populations due to differences in the severity of COVID-19, control measures, and socioeconomic contexts. However, the similar results in the proportions of the three groups with and without weighting (Table 1) and similar associations of the three groups with confidence in coping with the pandemic and mental health factors (Table 5) suggested that our results were not substantially affected by the demographic factors.

## Data availability statement

The datasets presented in this article are not readily available because our analysis and paper writing on the results are in progress. Requests to access the datasets should be directed to the corresponding author.

## Ethics statement

The studies involving human participants were reviewed and approved by the Institutional Review Board (IRB) of the University of Hong Kong Hospital Authority Hong Kong West Cluster (UW 20–651). The patients/participants provided their written informed consent to participate in this study.

## Author contributions

MPW, THL, SYH, and WG: conceptualization. MPW, WG, and YY: methodology and formal analysis. YY: writing–original draft preparation. WG, AL, SS, MPW, SYH, and THL: writing–review and editing. WG and MPW: supervision. MPW: project administration. All authors have read and agreed to the published version of the manuscript.

## Funding

This work was funded by the Hong Kong Jockey Club Charities Trust. The funder had no role in the design of the study; in the collection, analyses, or interpretation of data; in the writing of the manuscript, or in the decision to publish the results.

## Conflict of interest

The authors declare that the research was conducted in the absence of any commercial or financial relationships that could be construed as a potential conflict of interest.

## Publisher’s note

All claims expressed in this article are solely those of the authors and do not necessarily represent those of their affiliated organizations, or those of the publisher, the editors and the reviewers. Any product that may be evaluated in this article, or claim that may be made by its manufacturer, is not guaranteed or endorsed by the publisher.
